# Assessment of health-related quality of life in hypertensive hemodialysis patients

**DOI:** 10.7717/peerj.12690

**Published:** 2022-01-03

**Authors:** Ayesha Aslam, Shahid Shah, Ghulam Abbas, Anees ur Rehman, Tauqeer Hussain Malhi, Nasser Hadal Alotaibi, Abdulaziz Ibrahim Alzarea, Muhammad Fawad Rasool, Haris Khurram, Sibgha Noureen, Muhammad Talha Saeed Bokhari

**Affiliations:** 1 Department of Neurology, King Edward Medical University, Lahore, Pakistan; 2 Department of Pharmacy Practice, Faculty of Pharmaceutical Sciences, Government College University Faisalabad, Faisalabad, Pakistan; 3 Department of Pharmaceutics, Faculty of Pharmaceutical Sciences, Government College University Faisalabad, Faisalabad, Pakistan; 4 Department of Pharmacy Practice, Faculty of Pharmacy, Bahauddin Zakariya University, Multan, Pakistan; 5 Department of Clinical Pharmacy, College of Pharmacy, Jouf University, Sakaka, Al-Jouf Province, Kingdom of Saudi Arabia; 6 Department of Sciences and Humanities, National University of Computer and Emerging Sciences, Faisalabad, Pakistan

**Keywords:** Hypertension, Health related quality of life, Dialysis, Chronic kidney disease

## Abstract

**Background and Objectives:**

Globally, the prevalence of hypertension (HTN) with the coexistence of chronic kidney disease (CKD) is increasing, resulting in poor quality of life. The main objective of the study was to measure the health-related quality of life (HRQoL) of hypertensive hemodialysis patients.

**Methods:**

A multicenter follow-up study was carried out in six public and two private dialysis centers in Pakistan. A total of 517 hypertensive hemodialysis patients responded by completing the questionnaire at baseline and two subsequent phases. The quality of life of these patients was assessed using the EQ-5D-5L questionnaire (a standardized instrument for measuring generic health status). Statistical analysis was done using a multivariate linear regression model, Friedman test and Kruskal Wallis test.

**Results:**

The majority of patients (58.2%) had normal body mass index and about 60.5% of the patients were taking less salt due to HTN. Friedman test gave the statistically significant results (*p* ≤ 0.001) in systolic blood pressure (BP), diastolic BP and EQ-5D visual analogue scale (VAS) score between three phases (initial visit, first follow-up and second follow-up). A significant improvement was observed in self-care and usual activities from initial visit to first follow-up (*p* < 0.05). The most problematic dimension among the hypertensive patients with CKD was pain/discomfort (86.5%).

**Conclusions:**

HTN with coexisting CKD in hemodialysis patients severely affected HRQoL. Pain/discomfort was the most problematic dimension among the participants.

## Introduction

Hypertension (HTN) is one of the diseases that prevail every day in the world. According to the European Society of Cardiology and the European Society of Hypertension (ESC/ESH), HTN can be defined as blood pressure (BP) ≥ 140/80 mmHg (Folb et al., 2015). HTN can be a cause or a complication of chronic kidney disease (CKD) (Klag et al., 1996; George et al., 2016). Therefore, with its prevalence, the number of reported cases of CKD is also increasing. HTN affects 30% of the general adult population and up to 90% of people with CKD (Kearney et al., 2005; Muntner et al., 2010). According to the World Health Organization (WHO), HTN causes 9.4 million deaths worldwide (Savoia et al., 2017). In South Asia, there are about three times more likely to be hypertensive at a younger age than European whites (King-Shier et al., 2019).

The burden of CKD in underdeveloped Asian countries like Pakistan is increasing, where a considerable proportion of 180 million are prone to chronic disease like HTN and CKD (Jessani, Bux & Jafar, 2014). Studies from the past reveal that the patients suffering from HTN with coexistent CKD experience a marked decrease in health-related quality of life (HRQoL). HTN alone does not significantly affect HRQoL; the actual impairment in HRQoL occurs due to comorbidities such as CKD (Converse et al., 1992). The physical activities, mental health and mobility of the patient are all severely affected due to these complications which lead to impaired HRQoL (Theodorou et al., 2011). Worldwide, the effects of HTN with coexistent CKD on the social and psychological well-being of patients increased over time (Soni et al., 2010). Nowadays, HRQoL of patients is considered an important outcome of medical treatment (Baker, 1996). Furthermore, HRQoL is measured to assess the impact of the disease on the health of patients (McHorney, 1999). Many studies have reported on the HRQoL of HTN patients, but the combined effect of HTN and CKD on hemodialysis patient health is poorly understood (Kusek et al., 2002). It is therefore necessary to conduct a study to measure the HRQoL of these patients.

Over the past decades, the EuroQol 5-dimensional instrument (EQ-5D) has been recommended as a generic preference-based instrument for assessing the HRQoL in patients worldwide (Sullivan et al., 2020). However, its new version with a five level scale, namely EQ-5D-5L, was strongly recommended due to its reducing ceiling effect. The good validity and reliability of EQ-5D-5L in chronic diseases has been established (Dyer et al., 2010; Ascef et al., 2017; Huang et al., 2018; McClure et al., 2018). The relative effects of HTN and associated comorbidity on HRQoL using EQ-5D-5L in Pakistan have not been investigated. The objective of the study was to assess the HRQoL in hypertensive hemodialysis patients.

## Materials and Methods

### Study design and subjects

We carried out a multicenter follow-up study over a period of 2 months from January to February 2021. A stratified random sampling technique was used and the data were collected from the dialysis centers of 6 public hospitals and 2 private clinics in Pakistan with the help of physicians. The EQ-5D-5L was used as a generic instrument for describing and valuing health in terms of five dimensions: mobility, self-care, usual activities, pain/discomfort, and anxiety/depression. The questionnaire was given to the participants after being reviewed by three experts, two in cardiology and one in pharmacy education. For pilot study, the questionnaire was sent to 30 students to identify errors and misunderstood questions. The Cronbach’s alpha of the scores for the baseline was 0.72, while for the first follow-up it was 0.65 and for the second follow-up it was 0.61, indicating the consistency of the survey.

The EQ-5D-5L questionnaire was given to the participants to complete after having received an explanation of the objective of this study. An interview was conducted with written and verbal consent for non-educated participants after explanation of the study protocols. A socio-demographic questionnaire was also given to patients to collect general data from the patients which included, age, sex, marital status, employment status, smoking status, education, alcohol consumption, daily salt intake status (low salt intake < 5 g/day, normal salt intake = 5 g/day and high salt intake > 5 g/day), height and weight of patients and routine of exercise *etc*. A total of 517 patients had given the response. Heights and weights were taken to calculate body mass index (BMI) of the participants. After that the calculated BMI values were compared with the standard values. The patients having BMI < 18.5 kg/m^2^ were considered underweight and patients with BMI > 25 kg/m^2^ were considered overweight as the normal range of BMI is 18.5–24.5 kg/m^2^ (Theodorou et al., 2011). The other clinical factors like systolic BP and diastolic BP were calculated as well. The questionnaires were completed in three points: during the patient’s first visit and when enrolling in the study (phase A), 1 month after the first visit (phase B, first follow-up), and 2 months after the first visit (phase C, second follow-up).

### Ethics approval

The study design was approved by the Institutional Review Board of Government College University Faisalabad (GCUF/ERC/2438) which conforms to the Declaration of Helsinki. Each participant was interviewed privately to fill the questionnaires after taking informed consent form from them.

### Inclusion criteria

Hypertensive hemodialysis patients aged 18 years or older with systolic BP ≥140 mmHg were included in the study. The common etiology of CKD patients included in the study was diabetic nephropathy (36.2%), glomerulonephritis (28.4%), tubulo-interstitial disease (17.1%), renal stone disease (10.9%) and unknown cause (7.4%). All CKD patients included in the study had less than a month dialysis vintage.

### Exclusion criteria

Lactating patients, mentally ill patients or patients taking drugs of abuse like *Piper betle* (pan), *Areca catechu* (ghuttka), *Dalbergio sisso* (sheesha) were excluded from the study.

### Study variables

In our analysis, gender and age variables were considered as basic demographic characteristics (Tajima et al., 2010), while systolic BP and diastolic BP were considered as basic clinical features

### Quality of life instrument

The EQ-5D-5L instrument was used to measure the HRQoL of hypertensive hemodialysis patient. A multidisciplinary group of researchers with the collaboration of York University in 1987 designed this generic instrument (EuroqoQol, 2021). EQ-5D has support of two organizations the EuroQol Group Association and the EuroQol Research Foundation. The main purpose of using EQ-5D was to ensure high response rates due to its comprehensive and easily understandable feature. Moreover, its validity and reliability has been proven from the previous studies. The high correlation of the dimensions of this instrument with the dimensions of other instrument used widely has documented previously (Ghislandi et al., 2002).

The permission regarding use of required version of EQ-5D-5L was taken by filling the registration form available on EuroQol website. EQ-5D-5L consists of two pages, a descriptive page and a visual analogue scale (VAS). The descriptive page consists of five dimensions of health namely: mobility, self-care, usual activities, pain/discomfort and anxiety/depression. There are five different levels of each dimension namely: no problems, slight problems, moderate problems, serious problems and extreme problems (unable to do so) thus defining 3,125 different possible states of health.

The current health state was evaluated directly by using VAS. The VAS is basically a graduated scale (20 cm long) that is numbered from 0 to 100. 0 represents the worst possible health state while 100 represent the best possible health state. The respondents were asked to mark on the scale and the value obtained was taken as the quantitative measure of health outcome that the respondents’ own judgment represents. The value obtained from these two pages provided us the health status of a person. The questionnaire was filled by the patients according to their current situation and a five-digit value was obtained that was designated as the health state of that person at that time.

### Statistical analysis

Statistical Package for the Social Sciences (SPSS) Version 18.0, SPSS Inc., Chicago, Illinois, USA was used to analyze the data. Continuous and categorical data were collected where mean and standard deviation were used to express the continuous data and percentages for categorical data. Paired group differences between initial visits, first follow-up and second follow-up for different continuous variables were calculated by using Friedman test. Kruskal–Wallis tests were used to compare the categories of each socio-demographic characteristic for EQ-5D VAS score at initial visit, first follow-up and second follow-up. Multivariate regression model was used to evaluate the impact of socio-demographic and clinical variables on EQ-5D VAS score at initial visit, first and second follow-up. A two-tailed *p-value < 0.05* was considered statistically significant.

**Table 1:** Socio-demographic characteristics of study participants.

**Variables**	**Categories**	**Frequency (%)**
Gender	Female	232 (44.9)
	Male	285 (55.1)
Age	19–29	44 (8.5)
	30–39	114 (22.1)
	40–49	136 (26.3)
	50–59	117 (22.6)
	60–69	89 (17.2)
	70+	17 (3.3)
Employment status	Not capable	246 (47.6)
	No working	130 (25.1)
	Housewife	9 (1.7)
	Job/Labor	61 (11.8)
	Business	37 (7.2)
	Retired	28 (5.4)
	Missing	6 (1.2)
Marital status	Single	52 (10.1)
	Married	462 (89.4)
	Missing	3 (0.6)
Education	Not educated	104 (20.1)
	Primary	58 (11.2)
	Middle	101 (19.5)
	Secondary	156 (30.2)
	Higher secondary	48 (9.3)
	Graduate	50 (9.7)
Exercise	No exercise	350 (67.7)
	Sometimes	65 (12.6)
	Once a week	8 (1.5)
	Daily	91 (17.6)
	Missing	3 (0.6)
Salt intake	Low	313 (60.5)
	Normal	194 (37.5)
	High	2 (0.4)
	Missing	8 (1.5)
BMI^*^	Underweight	56 (10.8)
	Normal	301 (58.2)
	Overweight	114 (22.1)
	Obese	46 (8.9)
Family history of HTN	No	318 (61.5)
	Yes	199 (38.5)

**Notes.**

* BMI, Body mass index.

**Table 2:** Clinical data of the study participants.

Variables	Visits	Mean (S.D)	Median (Q_1_–Q_1_)	*p* ^a^
BMI	Initial visit	23.46 (4.5)	22.7 (20.2–26.2)	0.908
	First follow-up	23.53 (4.57)	22.7 (20.1–26.55)	
	Second follow-up	23.55 (4.52)	22.7 (20.5–26.4)	
Diastolic BP	Initial visit	85.07 (11.71)	85 (79–90)	0.001^*^
	First follow-up	84.99 (9.99)	85 (78–90)	
	Second follow-up	84.83 (10.35)	86 (78.5–90)	
Systolic BP	Initial visit	161.03 (16.58)	160 (150–170)	0.001^*^
	First follow-up	163.9 (15.8)	160 (151.5–170)	
	Second follow-up	159.42 (15.27)	156 (148–170)	
EQ-5D VAS score	Initial visit	56.51 (18.18)	55 (40–70)	0.001^*^
	first follow-up	57.95 (16.65)	55 (45–70)	
	Second follow-up	58.32 (17.12)	58 (45.5–70)	

**Notes.**

a Friedman test.

* *p* < 0.05.

S.D standard deviation BP blood pressure IQR interquartile range VAS visual analogue scale

## Results

A total of 517 patients responded successfully and completed EQ-5D questionnaire in all three stages (initial visit, first follow-up and second follow-up) of this study. Patient’s socio-demographic characteristics are presented in Table 1. The comparison of clinical data at three different stages (initial visit, first and second follow-up) of the patients that participated in this study has been shown in Table 2. There is no evidence of statistically significant difference in BMI at each stage. A statistically significant decrease was observed between the initial visit and the second follow-up with respect to diastolic BP. There was an increase in systolic BP at first stage and decrease in second stage resulting in the overall improvement. Similarly, a statistically significant improvement in EQ-5D VAS score was observed from initial visit to first follow-up and then from first follow-up to second follow-up (*p* ≤ 0.001) in all the statistically significant results.

Table 3 represents EQ-5D VAS score’s indexed quality of life by socio-demographic characteristics at baseline and follow-ups. In the gender variable, females tended to have higher VAS scores (57.56, 58.87 and 59.47 at initial visit, first and second follow-up respectively) as compared to males (55.66, 57.20 and 57.38 at initial visit, first and second follow-up respectively). The patients falling in the age category of 19–29 had the highest value of VAS scores (69.43, 69.11 and 69.66 at initial visit, first and second follow-up respectively) as compared to the rest age groups. The patients who didn’t exercise had the lowest VAS scores (54.08, 55.86 and 56.10 at initial visit, first and second follow-up respectively) while exercising patients had better scores as compared to them.

**Table 3:** Quality of life as indexed by EQ-5D VAS score at baseline and subsequent visits by socio-demographic characteristics.

Variables	Categories	Initial visit			First follow-up			Second follow-up		
		Mean (S.D)	Median (Q_1_–Q_3_)	*p* ^a^	Mean (S.D)	Median (Q_1_–Q_3_)	*P* ^a^	Mean (S.D)	Median (Q_1_–Q_3_)	*p* ^a^
Gender	Female	58.37 (20.12)	60 (40–70)	0.389	59.36 (18.45)	56 (45–74)	0.542	60.24 (18.56)	59 (46–70)	0.393
	Male	56.11 (16.4)	50 (44–70)		57.54 (15.02)	55 (50–65)		57.92 (15.65)	58 (45–70)		
Age	19–29	70.39 (17.3)	70 (52.5–85)	0.001	70.03 (14.99)	70 (50–85)	0.001	70.66 (16.77)	70 (50–87.5)	0.001
	30–39	60.81 (17.18)	60 (50–70)		61.59 (16.41)	60 (55–70)		62.18 (16.98)	60 (53–70)		
	40–49	57.7 (18.13)	60 (40–70)		58.84 (16.24)	60 (45–70)		59.83 (15.88)	60 (45–72)		
	50–59	56.3 (17.14)	50 (45–70)		56.81 (16.53)	55 (50–65)		56.98 (16.91)	55 (48–70)		
	60–69	47.89 (14.34)	50 (40–60)		50.84 (12.76)	50 (45–60)		51.41 (13.92)	50 (42–62)		
	70+	49.71 (22.46)	40 (35–70)		53.82 (21.25)	45 (35–75)		54.12 (20.02)	44 (40–73)		
Employment status	Not capable	51.16 (17.62)	50 (40–60)	0.001	53.69 (16.06)	50 (40–60)	0.001	53.84 (16.91)	50 (40–65)	0.001
	Not working	64.91 (16.8)	60 (50–80)		64.64 (14.83)	65 (55–75)		64.89 (16.13)	65 (52–75)		
	Housewife	60 (17.32)	60 (40–80)		62.67 (15.16)	63 (45–80)		65.33 (13)	66 (50–80)		
	Job/Labour	57.42 (16.88)	55 (45–70)		57.25 (17.33)	55 (45–65)		59.49 (15.28)	59 (48–70)		
	Business	66.77 (15.52)	70 (60–75)		67.13 (15.85)	70 (55–75)		67.19 (15.16)	72 (57–75)		
	Retired	60.18 (15.6)	60 (50–70)		60.68 (15.21)	57.5 (50–73)		62.64 (14.73)	61 (54–75)		
Marital status	Single	64.35 (18.9)	60 (50–82.5)	0.002	64.37 (16.67)	65 (50–80)	0.006	65.48 (19.39)	65 (50–84)	0.004
	Married	56.25 (17.84)	52.5 (40–70)		57.64 (16.45)	55 (45–70)		58.17 (16.54)	57 (45–70)		
Education	Not Educated	53.23 (18.09)	50 (37.5–70)	0.009	54.92 (16.39)	54 (40–65)	0.005	56.19 (16.72)	54 (40–70)	0.030
	Primary	53.6 (22.38)	50 (40–60)		54.92 (20.43)	55 (40–65)		54.4 (21.06)	52.5 (42–68)		
	Middle	62.67 (19.42)	60 (45–80)		63.74 (18.68)	65 (50–80)		63.48 (18.39)	65 (50–75)		
	Secondary	54.58 (15.41)	50 (45–70)		56.42 (13.46)	55 (50–65)		57 (14.57)	57.5 (50–70)		
	Higher secondary	61.5 (12.49)	60 (50–70)		60.69 (13.01)	60 (47.5–70)		61.9 (11.88)	62.5 (50–70)		
	Graduate	62.05 (18.16)	60 (50–70)		62.69 (15.89)	55 (50–75)		64.28 (17.36)	56 (48–73)		
Exercise	No exercise	54.17 (17.66)	50 (40–70)	0.001	55.8 (16.41)	55 (45–65)	0.001	56.18 (16.8)	54 (45–70)	0.001
	Sometimes	61.23 (21.3)	60 (40–80)		62.61 (19.01)	55 (50–80)		63.11 (20.05)	60 (50–80)		
	Once a week	68.33 (15.71)	60 (50–77.5)		68.33 (14.38)	62.5 (50–77.5)		67.67 (14.04)	61.5 (50–76.5)		
	Daily	64.5 (14.31)	60 (50–70)		64.26 (12.97)	65 (55–75)		65.81 (12.26)	66 (53–73)		
Salt intake	Low	56.55 (18.18)	60 (40–70)	0.433	58.09 (17.11)	55 (45–70)	0.604	57.96 (17.43)	57 (45–70)	0.231
	Normal	57.94 (17.97)	60 (45–70)		58.71 (15.69)	55 (50–70)		60.48 (16.11)	60 (48–72)		
	High	50.00	50 (50–50)		50.00	50 (50–50)		50.00	50 (50–50)		
BMI	Underweight	52.94 (18.75)	60 (40–75)	0.454	55.15 (15.4)	55 (40–70)	0.356	55.08 (17.06)	55 (40–73)	0.661
	Normal	57.25 (16.5)	55 (40–70)		58.94 (14.85)	53.5 (50–65)		59.07 (15.72)	52.5 (47–70)		
	Overweight	58.2 (20.34)	60 (40–70)		58.93 (19.5)	56 (45–74)		60.3 (18.4)	59 (46–70)		
	Obese	58 (21.33)	50 (44–70)		57.18 (20)	55 (50–65)		59.51 (20.06)	58 (45–70)		
Family history of HTN	No	56.95 (18.14)	50 (40–60)	0.876	58.27 (17.27)	55 (45–60)	0.820	59.27 (17.07)	58 (42–64)	0.411
	Yes	57.27 (18.08)	55 (45–70)		58.41 (15.47)	55 (50–70)		58.33 (16.85)	60 (50–70)		

**Notes.**

a Kruskal Wallis Test, HTN is hypertension.

The patients who took normal quantity of salt in their diet showed the highest VAS values with 58.04, 58.79 and 60.22 at initial visit, first and second follow-up respectively and people taking high amount of salt in their diet showed lowest VAS scores and that value remained constant in the latter two stages of study as well. The patients with no family history of HTN had a slightly better VAS score as compared to persons having family history of HTN. The persons with normal BMI showed the highest VAS values and the mean values obtained at the initial visit, first and second follow-up were 56.86, 58.89 and 59.01 respectively. Patients falling in underweight category had the lowest VAS values and the mean values obtained were 52.89, 55.49 and 55.58 at initial visit, first and second follow-up respectively. As it is seen from Table 3, there are no significant differences in VAS scores depending on salt intake, BMI and HTN family history. And such tendency is observed in all three visits.

Multivariate linear regression analysis of EQ-5D-VAS score and variables (socio-demographic and clinical) are shown in Table 4. EQ-5D VAS scores in each stage are considered as dependent variable and, socio-demographic and clinical variables are considered as predictors. Meanwhile, *p* < 0.05 shows the statistically significant effect and contribution of the coefficient of each category and variable in EQ-5D VAS score. The *p-value* of the 19–29 and 30–39 age categories had a significant positive effect on the EQ-5D-VAS score at the initial visit, the first follow-up and the second follow-up. Respondents who were not able to work or do a job/job and who didn’t exercise had a lower HRQoL than others. While respondents who consumed little salt had a good HRQoL compared to others. BMI had a significant positive while HTN had a significant negative effect on HRQoL.

**Table 4:** Multivariate linear regression analyses of EQ-5D- VAS score and socio-demographic and clinical variables.

Parameters	Categories	Initial visit		First follow-up		Second follow-up	
		Coefficient	*p*	Coefficient	*p*	coefficient	*p*
Intercept		34.864	0.031	27.789	0.055	77.385	0.001
Gender	Female	3.173	0.056	2.667	0.087	4.918	0.004
	Male	0^a^	.	0^a^	.	0^a^	.
Age	19–29	20.848	0.001	17.024	0.001	17.843	0.001
	30–39	11.867	0.011	8.436	0.053	10.899	0.015
	40–49	6.595	0.148	4.027	0.344	6.233	0.158
	50–59	5.132	0.242	1.856	0.652	5.541	0.190
	60–69	0.580	0.897	−1.700	0.687	0.999	0.815
	70+	0^a^	.	0^a^	.	0^a^	.
Employment status	Not capable	−9.161	0.012	−7.933	0.020	−8.136	0.019
	Not working	1.206	0.766	−0.068	0.986	−2.057	0.601
	Housewife	−3.631	0.594	0.251	0.969	−2.058	0.750
	Job/Labour	−7.961	0.056	−9.506	0.015	−9.085	0.022
	Business	3.687	0.417	3.854	0.369	2.618	0.552
	Retired	0^a^	.	0^a^	.	0^a^	.
Marital status	Single	−3.338	0.288	−3.656	0.217	−2.637	0.379
	Married	0^a^	.	0^a^	.	0^a^	.
Education	Not educated	−2.620	0.423	−2.644	0.383	−4.649	0.150
	Primary	−3.609	0.299	−4.002	0.221	−8.177	0.019
	Middle	1.892	0.553	1.959	0.512	−0.021	0.995
	Secondary	−2.262	0.439	−0.965	0.723	−5.716	0.049
	Higher secondary	4.209	0.236	1.957	0.558	1.204	0.731
	Graduate	0^a^	.	0^a^	.	0^a^	.
Exercise	No exercise	−7.406	0.001	−5.634	0.004	−8.904	0.001
	Sometimes	−2.991	0.283	−1.377	0.598	−3.363	0.226
	Once a week	7.054	0.330	6.952	0.305	15.712	0.058
	Daily	0^a^	.	0^a^	.	0^a^	.
Salt intake	Low	29.994	0.013	27.008	0.017	−2.912	0.055
	Normal	32.109	0.008	28.219	0.013	0^a^	.
	High	0^a^	.	0^a^	.		
BMI		0.552	0.001	0.338	0.027	0.337	0.051
Systolic blood pressure		−0.075	0.118	0.070	0.239	−0.066	0.215
Diastolic blood pressure		−0.029	0.675	−0.101	0.267	−0.038	0.629
Hypertension		−0.023	0.043	−0.027	0.012	−0.040	0.001

**Figure 1:**
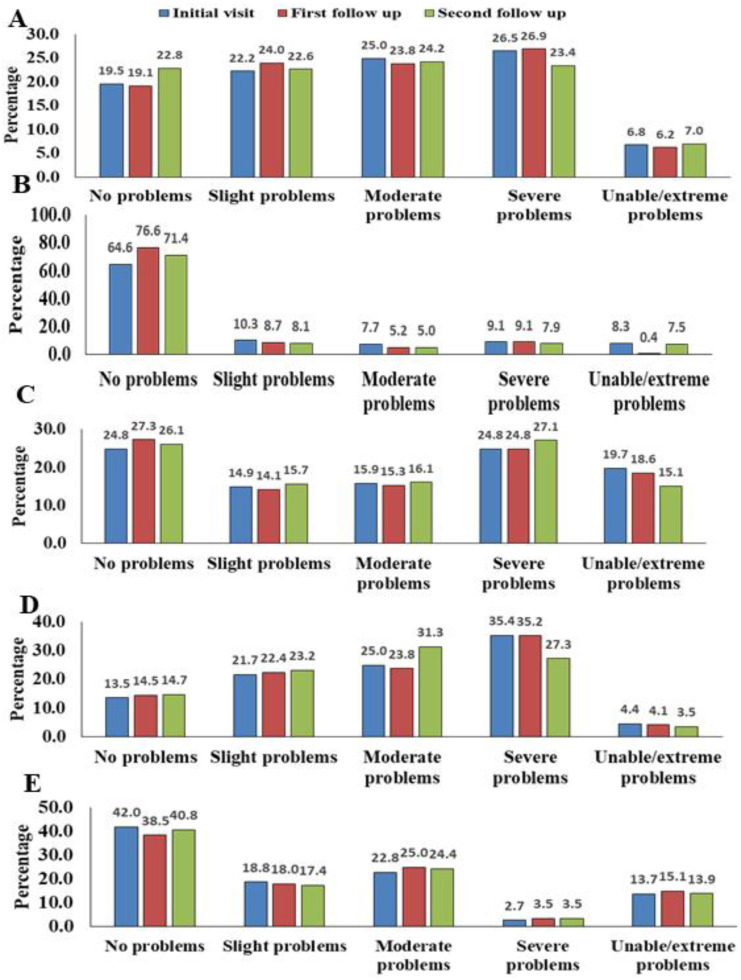
Percentages of participants with regard to EQ-5D-5L dimensions and levels reported the degree of severity. (A) Mobility, (B) self-care, (C) usual activity, (D) pain/discomfort and (E) anxiety/depression.

Percentages of participants with regard to EQ-5D-5L dimensions and levels reporting the degree of severity are presented in Figs. 1A–1E (graphs). 5 different levels including “no problems”, “slight problems”, “moderate problems”, “severe problems” and “extreme problems” were used to represent each of the dimensions including mobility, self-care, casual activity, pain/discomfort and anxiety/depression.

The most frequently observed problematic dimension was pain and discomfort. Almost 86.5% of patients experienced pain and discomfort during the first visit. This percentage declined to 85.5% then to 85.3% in the following two phases (first and second follow-up) respectively. In the self-care dimension, 35.4% of patients reported management problems at the time of the initial visit. This percentage decreased to 24.4%, then to 28.6% on the first and second follow-up, respectively showing a positive overall response.

Concerning the fifth level of severity (“extreme problems”) the following results were observed. In mobility, 6.8% patients were noted that were unable to move and this value decreased to 6.2% and then increased to 7% for the next two stages (first and second follow-up). In self-care dimension, 8.3% patients were not able to wash or dress themselves at initial visit and this value declined to 0.4% at the first follow-up and then rose to 7.5% during second follow-up. In usual activities dimension there was a marked improvement noticed in level five values. 19.7% of the patients were unable to do their usual activities at the initial visit and that value decreased to 18.6% and 15.1% in the next two phases (1st and 2nd follow-up) respectively. 4.4% patients were in extreme pain or discomfort and this number decreased to 4.1% at the first follow-up and 3.5% at the second follow-up. In anxiety/depression, 13.7% patients were extremely anxious or depressed at initial phase and this value rose to 15.1% and then decreased to 13.9% in the next two phases (first and second follow-up) respectively. Majority of patients had no problem with the dimension of self-care (64.6%, 76.6% and 71.4% in the three phases of the study respectively).

## Discussion

HRQoL is often considered to be equal, if not more important than quantity of life. Our results showed significant improvement in the systolic BP, diastolic BP and EQ-5D VAS score from initial visit to first follow-up and second follow-up respectively *(p* ≤ *0.001)*. It was due to the fact that majority of the patients (60.5%) were taking less salt in their diet and were following the instructions of physician after consultation. In our study, the most frequently observed problematic dimension of EQ-5D-5L was pain/discomfort and the dimensions like self-care and usual activities were least affected. These results are consistent with the recent studies conducted in China (Liang et al., 2019), Bulgaria {Encheva, 2020 #14} (Encheva et al., 2020) and Trinidad and Tobago (Bailey et al., 2019) where the problematic dimension was also pain/discomfort). Our study contradicted that of Wang et al. (2009), who reported HTN association with the dimension anxiety/depression. This might be due to the fact that we included hypertensive hemodialysis patients with CKD than HTN alone. It showed that co-morbidities along with HTN had a severe effect on HRQoL.

In our study, the EQ-5D VAS score was higher in women than in men. These results contradict a cross-sectional study of African Americans with chronic insufficiency using 36-Item Short-Form (SF-36) (Kusek et al., 2002) and a study of Bulgarian population norms (Encheva et al., 2020) where the results were significantly higher in men than women. These inconsistent results could be due to changes in geographical regions and the climatic conditions.

Hypertensive patients are usually cautioned not to limit physical activity to avoid worsening of HTN (Bakker et al., 2018). However, our study showed that hypertensive hemodialysis patients with concomitant CKD limited their mobility and physical activities, because renal dysfunction in patients with CKD might prevent physical activities and lower the HRQoL. Finally, the quality of life of participants indexed by EQ-5D-VAS score was observed according to their socio-demographics, and statistically significant results were obtained in the majority of variables namely; age, employment status, marital status, education and exercise (*p* < 0.05). In the same way, multivariate linear regression analyses of EQ-5D- VAS score at different phases showed significant results for socio-demographic and clinical variables. It shows that the HTN with renal failure is associated with all these variables that affect the quality of life of the patients*.* These results are consistent with the results of Khosravi et al. (2010) where the education, income, occupation and marital status were all associated with quality of life of hypertensive patients. Our data is also compatible to the results of African American study with CKD where employment status, education level, BMI, exercise status, age and marital status showed statistically significant results (Kusek et al., 2002). It is recognized that socio-economic factors have a significant influence on following physician’s instructions in hypertensive patients (Czarnecka et al., 2006); therefore, these results may be partly ascribing to increased treatment adherence among some groups of participants. We found the reduced HRQoL among the hypertensive patients in pain/discomfort (4th dimension of EQ-5D-5L) to the great extent due to coexistent CKD. Our study found that low salt-intake, regular exercise and maintaining a standard BMI were the main factors for good HRQoL.

Our study is limited because the questionnaire used to collect data from patients only asks patients what they are feeling today. Therefore, sudden episodes of pain or discomfort can lead to an inappropriate response. As the questionnaire is a self-assessment, patients can report high HRQoL when they adjust to the symptoms of the disease.

## Conclusion

HTN with coexistent CKD severely affects HRQoL. Pain/discomfort is the most severely affected dimension in participants. Following the dialysis schedule, the proper treatment of HTN and physician consultation are the key strategies to maintain the HRQoL of these patients. A good management of the disease may improve the quality of life of patient that can be preferably checked by EQ-5D-5L.

## Supplemental Information

10.7717/peerj.12690/supp-1 Supplemental Information 1 Raw data

10.7717/peerj.12690/supp-2 Supplemental Information 2 Statistical reporting

10.7717/peerj.12690/supp-3 Supplemental Information 3 Questionnaire

## Additional Information and Declarations

### Data Availability

The following information was supplied regarding data availability: The raw data is available in the Supplemental File.
