# Supplementary material for: Assessment of health-related quality of life in hypertensive hemodialysis patients
Source: PeerJ. 2022 Jan 3;10:e12690. doi: 10.7717/peerj.12690 (PMC8734469; doi:10.7717/peerj.12690)
Supplement: Supplemental Information 2 [file peerj-10-12690-s002.docx]

**Health**

| Parameter | B | Std. Error | t | *p*-value |
| --- | --- | --- | --- | --- |
|  |  |  |  |  |
| Intercept | 34.864 | 16.125 | 2.162 | .031 |
| [Gender=0] | 3.173 | 1.659 | 1.912 | .056 |
| [Gender=1] | 0^a^ | . | . | . |
| [Age=1] | 20.848 | 5.499 | 3.791 | .000 |
| [Age=2] | 11.867 | 4.640 | 2.558 | .011 |
| [Age=3] | 6.595 | 4.546 | 1.451 | .148 |
| [Age=4] | 5.132 | 4.378 | 1.172 | .242 |
| [Age=5] | .580 | 4.471 | .130 | .897 |
| [Age=6] | 0^a^ | . | . | . |
| [EmploymentStatus=0] | -9.161 | 3.641 | -2.516 | .012 |
| [EmploymentStatus=1] | 1.206 | 4.041 | .298 | .766 |
| [EmploymentStatus=2] | -3.631 | 6.803 | -.534 | .594 |
| [EmploymentStatus=3] | -7.961 | 4.160 | -1.914 | .056 |
| [EmploymentStatus=4] | 3.687 | 4.541 | .812 | .417 |
| [EmploymentStatus=5] | 0^a^ | . | . | . |
| [MaritalStatus=0] | -3.338 | 3.141 | -1.063 | .288 |
| [MaritalStatus=1] | 0^a^ | . | . | . |
| [Education=0] | -2.620 | 3.269 | -.802 | .423 |
| [Education=1] | -3.609 | 3.475 | -1.039 | .299 |
| [Education=2] | 1.892 | 3.186 | .594 | .553 |
| [Education=3] | -2.262 | 2.921 | -.775 | .439 |
| [Education=4] | 4.209 | 3.547 | 1.187 | .236 |
| [Education=5] | 0^a^ | . | . | . |
| [SmokingStatus=1] | -3.115 | 2.187 | -1.425 | .155 |
| [SmokingStatus=2] | -7.729 | 4.363 | -1.771 | .077 |
| [SmokingStatus=3] | 0^a^ | . | . | . |
| [Exercise=0] | -7.406 | 2.045 | -3.621 | .000 |
| [Exercise=1] | -2.991 | 2.781 | -1.075 | .283 |
| [Exercise=2] | 7.054 | 7.234 | .975 | .330 |
| [Exercise=3] | 0^a^ | . | . | . |
| [SaltIntake=1] | 29.994 | 12.042 | 2.491 | .013 |
| [SaltIntake=2] | 32.109 | 12.058 | 2.663 | .008 |
| [SaltIntake=3] | 0^a^ | . | . | . |
| BMI | .552 | .166 | 3.334 | .001 |
| SystolicBloodPressure | -.075 | .048 | -1.568 | .118 |
| DiastolicBloodPressure | -.029 | .069 | -.420 | .675 |
| Hypertension | -.023 | .012 | -2.034 | .043 |
| Dialysis | .000 | .000 | .994 | .321 |

**F1 Health**

| Parameter | B | Std. Error | t | *p*-value |
| --- | --- | --- | --- | --- |
|  |  |  |  |  |
| Intercept | 27.789 | 14.419 | 1.927 | .055 |
| [Gender=0] | 2.667 | 1.556 | 1.714 | .087 |
| [Gender=1] | 0^a^ | . | . | . |
| [Age=1] | 17.024 | 5.120 | 3.325 | .001 |
| [Age=2] | 8.436 | 4.340 | 1.944 | .053 |
| [Age=3] | 4.027 | 4.254 | .947 | .344 |
| [Age=4] | 1.856 | 4.112 | .451 | .652 |
| [Age=5] | -1.700 | 4.220 | -.403 | .687 |
| [Age=6] | 0^a^ | . | . | . |
| [EmploymentStatus=0] | -7.933 | 3.407 | -2.328 | .020 |
| [EmploymentStatus=1] | -.068 | 3.788 | -.018 | .986 |
| [EmploymentStatus=2] | .251 | 6.349 | .039 | .969 |
| [EmploymentStatus=3] | -9.506 | 3.880 | -2.450 | .015 |
| [EmploymentStatus=4] | 3.854 | 4.285 | .899 | .369 |
| [EmploymentStatus=5] | 0^a^ | . | . | . |
| [MaritalStatus=0] | -3.656 | 2.957 | -1.236 | .217 |
| [MaritalStatus=1] | 0^a^ | . | . | . |
| [Education=0] | -2.644 | 3.027 | -.873 | .383 |
| [Education=1] | -4.002 | 3.265 | -1.225 | .221 |
| [Education=2] | 1.959 | 2.989 | .656 | .512 |
| [Education=3] | -.965 | 2.718 | -.355 | .723 |
| [Education=4] | 1.957 | 3.338 | .586 | .558 |
| [Education=5] | 0^a^ | . | . | . |
| [SmokingStatus=1] | -1.891 | 2.049 | -.923 | .357 |
| [SmokingStatus=2] | -4.764 | 4.104 | -1.161 | .246 |
| [SmokingStatus=3] | 0^a^ | . | . | . |
| [Exercise=0] | -5.634 | 1.927 | -2.924 | .004 |
| [Exercise=1] | -1.377 | 2.610 | -.528 | .598 |
| [Exercise=2] | 6.952 | 6.766 | 1.028 | .305 |
| [Exercise=3] | 0^a^ | . | . | . |
| [SaltIntake=1] | 27.008 | 11.294 | 2.391 | .017 |
| [SaltIntake=2] | 28.219 | 11.322 | 2.492 | .013 |
| [SaltIntake=3] | 0^a^ | . | . | . |
| Hypertension | -.027 | .011 | -2.510 | .012 |
| Dialysis | .000 | .000 | .928 | .354 |
| F1Systolic | .070 | .059 | 1.180 | .239 |
| F1Diastolic | -.101 | .091 | -1.111 | .267 |
| F1BMI | .338 | .152 | 2.225 | .027 |

**F2 Health**

| Parameter | B | Std. Error | t | *p-*value |
| --- | --- | --- | --- | --- |
|  |  |  |  |  |
| Intercept | 77.385 | 11.350 | 6.818 | .000 |
| [Gender=0] | 4.918 | 1.680 | 2.928 | .004 |
| [Gender=1] | 0^a^ | . | . | . |
| [Age=1] | 17.843 | 5.332 | 3.346 | .001 |
| [Age=2] | 10.899 | 4.476 | 2.435 | .015 |
| [Age=3] | 6.233 | 4.409 | 1.414 | .158 |
| [Age=4] | 5.541 | 4.218 | 1.314 | .190 |
| [Age=5] | .999 | 4.270 | .234 | .815 |
| [Age=6] | 0^a^ | . | . | . |
| [EmploymentStatus=0] | -8.136 | 3.460 | -2.351 | .019 |
| [EmploymentStatus=1] | -2.057 | 3.926 | -.524 | .601 |
| [EmploymentStatus=2] | -2.058 | 6.462 | -.318 | .750 |
| [EmploymentStatus=3] | -9.085 | 3.963 | -2.292 | .022 |
| [EmploymentStatus=4] | 2.618 | 4.400 | .595 | .552 |
| [EmploymentStatus=5] | 0^a^ | . | . | . |
| [MaritalStatus=0] | -2.637 | 2.995 | -.881 | .379 |
| [MaritalStatus=1] | 0^a^ | . | . | . |
| [Education=0] | -4.649 | 3.221 | -1.443 | .150 |
| [Education=1] | -8.177 | 3.468 | -2.358 | .019 |
| [Education=2] | -.021 | 3.146 | -.007 | .995 |
| [Education=3] | -5.716 | 2.890 | -1.978 | .049 |
| [Education=4] | 1.204 | 3.495 | .345 | .731 |
| [Education=5] | 0^a^ | . | . | . |
| [SmokingStatus=1] | -1.424 | 2.108 | -.675 | .500 |
| [SmokingStatus=2] | -3.181 | 4.457 | -.714 | .476 |
| [SmokingStatus=3] | 0^a^ | . | . | . |
| [Exercise=0] | -8.904 | 2.041 | -4.363 | .000 |
| [Exercise=1] | -3.363 | 2.773 | -1.212 | .226 |
| [Exercise=2] | 15.712 | 8.279 | 1.898 | .058 |
| [Exercise=3] | 0^a^ | . | . | . |
| [SaltIntake=1] | -2.912 | 1.510 | -1.928 | .055 |
| [SaltIntake=2] | 0^a^ | . | . | . |
| hypertension | -.040 | .011 | -3.485 | .001 |
| dialysis | .001 | .000 | 1.502 | .134 |
| F2Systolic | -.066 | .053 | -1.243 | .215 |
| F2Diastolic | -.038 | .079 | -.484 | .629 |
| F2BMI | .337 | .172 | 1.961 | .051 |
