# Supplementary material for: Assessment of health-related quality of life in hypertensive hemodialysis patients
Source: PeerJ. 2022 Jan 3;10:e12690. doi: 10.7717/peerj.12690 (PMC8734469; doi:10.7717/peerj.12690)
Supplement: Supplemental Information 3 [file peerj-10-12690-s003.docx]

**Assessment of Health Related Quality of Life in Hypertensive Patients with Renal Failure**

1. **Patient name -------------------------------**
2. **Gender**
3. Male
4. Female
5. **Patient age ---------**
6. **Patient weight ---------**
7. **Patient height ---------**
8. **Employment status**
9. Not capable of working
10. Retired
11. Private business
12. Doing a job
13. not working
14. **Marital status**
15. Married
16. Widowed
17. single
18. **Education**
19. Primary
20. Middle
21. Secondary
22. Higher secondary
23. Graduation
24. Not educated
25. **Smoking status**
26. Non smoker
27. Ex-smoker
28. Smokers
29. **Exercise**
30. Daily
31. Once a week
32. Sometimes
33. No exercise
34. **Alcohol consumption**
35. No
36. < 1 glass/ day
37. ≥ 1 glass / day
38. **Risk group**
39. CVD (cardiovascular disease)
40. DM (diabetes mellitus)
41. Hepatitis
42. Any other

**14. Salt intake**

1. Low
2. Normal
3. High

**Clinical data**

1. BMI (body mass index) -----------
2. Systolic pressure ----------
3. Diastolic pressure -----------

**When you were diagnosed with hypertension?**

**-------------------**

**When dialysis was initiated?**

**-------------------**

**Family history of HTN**

**-------------**

Under each heading, please tick the ONE box that best describes your health TODAY.

# MOBILITY

I have no problems in walking about 

I have slight problems in walking about 

I have moderate problems in walking about 

I have severe problems in walking about 

I am unable to walk about 

# SELF-CARE

I have no problems washing or dressing myself 

I have slight problems washing or dressing myself 

I have moderate problems washing or dressing myself 

I have severe problems washing or dressing myself 

I am unable to wash or dress myself 

**USUAL ACTIVITIES** *(e.g. work, study, housework, family or leisure activities)*

I have no problems doing my usual activities 

I have slight problems doing my usual activities 

I have moderate problems doing my usual activities 

I have severe problems doing my usual activities 

I am unable to do my usual activities 

# PAIN / DISCOMFORT

I have no pain or discomfort 

I have slight pain or discomfort 

I have moderate pain or discomfort 

I have severe pain or discomfort 

I have extreme pain or discomfort 

# ANXIETY / DEPRESSION

I am not anxious or depressed 

I am slightly anxious or depressed 

I am moderately anxious or depressed 

I am severely anxious or depressed 

I am extremely anxious or depressed 

- We would like to know how good or bad your health is TODAY.
- This scale is numbered from 0 to 100.
- 100 mean the best health you can imagine. 0 means the worst health you can imagine.
- Mark an X on the scale to indicate how your health is TODAY.
- Now, please write the number you marked on the scale in the box below.

The best health you can imagine

100

95

90

85

80

75

70

65

60

55

YOUR HEALTH TODAY = 50

45

40

35

30

25

20

15

10

5

0

The worst health you can imagine
